# Association between impaired sensitivity to thyroid hormones and sedentary behavior: a cross-sectional study

**DOI:** 10.3389/fmed.2025.1596669

**Published:** 2025-06-06

**Authors:** Hangzhou Yang, Jie Kang, Lingkang Dong, Zihan Lin, Qixian Lin, Bo Wu

**Affiliations:** ^1^Department of General Surgery, Shanghai Sixth People's Hospital Affiliated to Shanghai Jiao Tong University School of Medicine, Shanghai, China; ^2^Department of Otolaryngology Head and Neck Surgery, Shanghai Sixth People's Hospital Affiliated to Shanghai Jiao Tong University School of Medicine, Shanghai, China

**Keywords:** NHANES, sedentary, PTFQI, sensitivity to thyroid hormones, thyroid hormone

## Abstract

**Purpose:**

Sedentary behavior and impaired thyroid hormone sensitivity are linked to a variety of comorbid conditions; however, the exact nature of their relationship remains inadequately studied. This study sought to examine the association between sedentary time and thyroid hormone sensitivity.

**Methods:**

Utilizing a cross-sectional design, the study analyzed data from U.S. participants in the National Health and Nutrition Examination Survey (NHANES) conducted between 2007 and 2012. The Least Absolute Shrinkage and Selection Operator (LASSO) regression and the Boruta algorithm were employed to screen out confounding factors closely associated with sedentary time and the parametric thyroid feedback quantile-based index (PTFQI). Multivariate linear regression models were applied to analyze the association between sedentary time and indicators of thyroid hormone sensitivity. After adjusting for all confounding factors, restricted cubic spline (RCS) curves were utilized to further explore the potential non-linear relationship between sedentary time and indicators of thyroid hormone sensitivity. Additionally, subgroup analyses and interaction tests were conducted to further explore this association.

**Results:**

A total of 22 significant confounding factors were identified through LASSO regression and the Boruta algorithm. Among these potential confounding factors, body mass index (BMI) occupied a central position, and it partially mediated the association between sedentary time and the PTFQI. RCS analysis indicated that, after adjusting for all covariates, there was a significant linear association between sedentary time and PTFQI in men (*P* for overall = 0.002, *P* for non-linear = 0.085). In contrast, in women, the relationship presented an “inverted U-shaped” curve, which was not statistically significant (*P* for overall > 0.05). Moreover, the results of the interaction analysis revealed a significant interaction effect of race on the association between sedentary time and PTFQI (*P* for interaction = 0.004).

**Conclusions:**

In this study, we found a positive association between impaired thyroid hormone sensitivity and sedentary time in men after adjusting for confounders, and BMI partially mediated this positive association. Additionally, the factor of race exhibited a significant interaction effect on the association between sedentary time and the PTFQI.

## Introduction

In both developed and developing nations, social, economic, and environmental transitions have precipitated a pervasive rise in sedentary behavior (SB) (1). Sedentary behavior (SB) has emerged as a prominent aspect of contemporary life, garnering considerable attention due to its implications for health (2). The physical activity (PA) guidelines promulgated by the World Health Organization in 2020 define sedentary behavior as “any behavior in a sitting, reclining, or lying position during wakefulness, with an energy expenditure of 1.5 metabolic equivalents or less” encompassing activities such as prolonged sitting at work, television viewing, and the use of electronic devices (3).

Thyroid hormones play a crucial role in regulating the body's metabolism and energy expenditure and are key factors in maintaining metabolic homeostasis. Thyroid dysfunction is closely associated with the occurrence of various metabolic diseases (4). Impaired thyroid hormone sensitivity refers to a weakened response of the body to thyroid hormones, which may manifest as resistance to or a decrease in sensitivity to the effects of thyroid hormones, thus having a wide-ranging impact on an individual's health. Previous research has demonstrated that thyroid-stimulating hormone (TSH) levels alone do not provide an objective measure of thyroid function, as TSH secretion is inversely regulated by peripheral free thyroxine (FT4) concentrations (5). Quantitative indicators such as thyrotrophin thyroxine resistance index (TT4RI), thyroid-stimulating hormone index (TSHI), and parametric thyroid feedback quantile-based index (PTFQI) are employed to assess the sensitivity of the pituitary-central axis to thyroid hormones (6–8). The PTFQI, in particular, utilizes a mathematical model incorporating TSH and FT4 levels to reflect the feedback regulation state of the pituitary-thyroid axis, thereby quantifying thyroid hormone sensitivity. Compared to TSHI and TT4RI, PTFQI offers a more precise quantification of changes in thyroid hormone sensitivity and demonstrates superior stability (9, 10).

**Figure 1:**
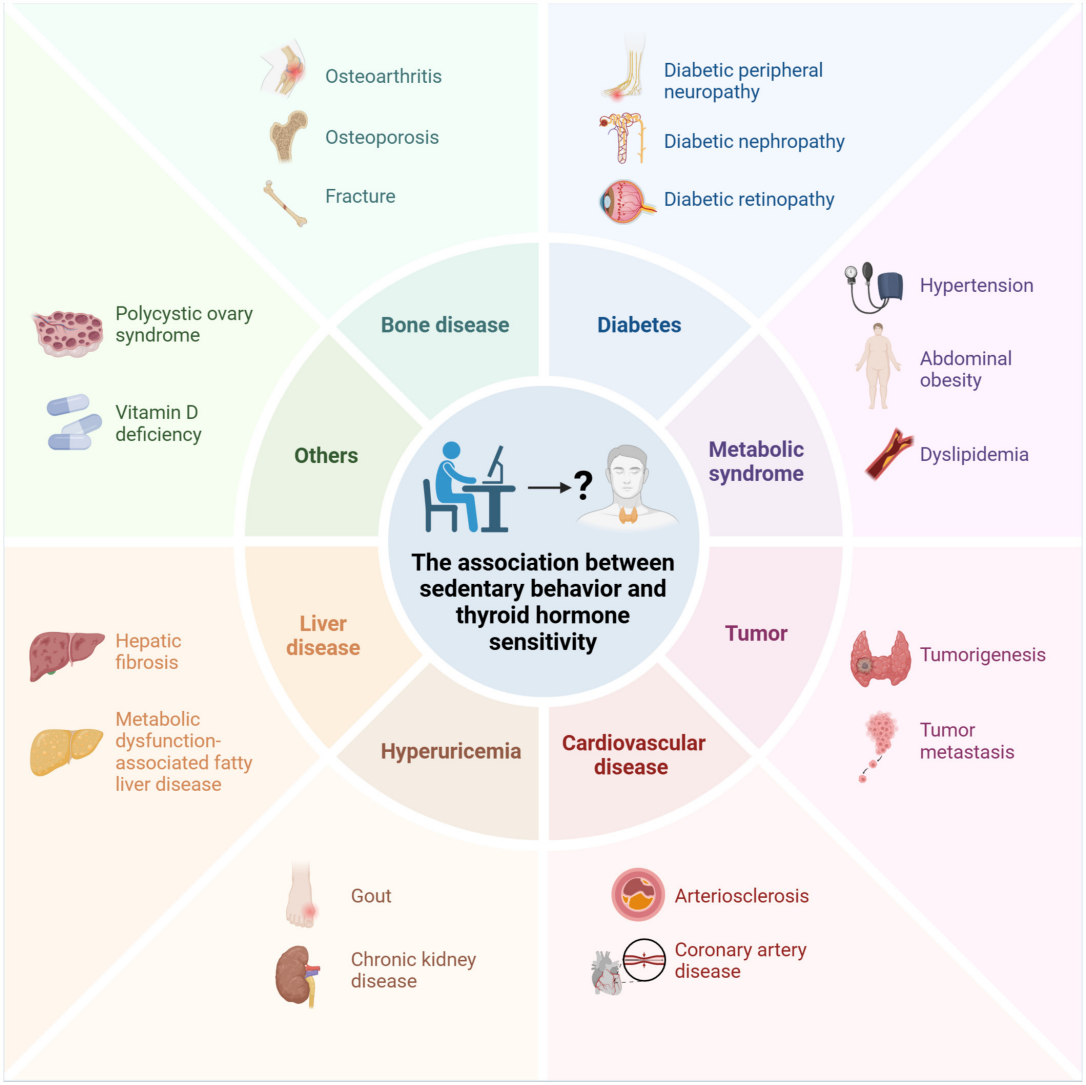
Sedentary behavior is associated with a series of comorbid conditions related to impaired thyroid hormone sensitivity.

The interrelationship between sedentary behavior and impaired thyroid hormone sensitivity encompasses a range of comorbidities (Figure 1). Contemporary research has substantiated that sedentary behavior serves as a significant catalyst for metabolic and endocrine disorders, with its detrimental effects on public health warranting considerable attention (11). Sedentary behavior is particularly associated with an increased incidence of cardiovascular diseases (12, 13), liver diseases (14, 15), kidney diseases (16, 17), tumorigenesis and tumor progression (18, 19), obesity (20), compromised bone health (21, 22), and metabolic syndrome (23). Concurrently, impaired thyroid hormone sensitivity is intricately linked to metabolic syndrome, hypertension, hyperuricemia, vitamin D deficiency, diabetes, and related conditions (24–28). Sun et al. (29) have demonstrated that reduced sensitivity to thyroid hormones in individuals with subclinical hypothyroidism is linked to an elevated risk of cardiovascular disease and obesity. Among elderly individuals with normal thyroid function, diminished thyroid hormone sensitivity is correlated with an increased incidence of osteoporosis and fractures, independent of traditional risk factors (30, 31). Furthermore, impaired sensitivity to thyroid hormones is associated with a heightened risk of thyroid cancer and cervical lymph node metastasis (32, 33).

Nevertheless, the relationship between impaired thyroid hormone sensitivity and sedentary behavior remains inadequately understood. Consequently, this study utilized data from the National Health and Nutrition Examination Survey (NHANES) spanning 2007–2012 to investigate the independent association between thyroid hormone sensitivity and sedentary behavior.

## Materials and methods

### Study population

The NHANES is a nationally representative survey employing a multi-stage, stratified sampling methodology. Ethical approval for the NHANES study was obtained from the Institutional Review Board of the National Center for Health Statistics (NCHS) in the United States, with all participants providing informed consent prior to participation. This study utilized NHANES data from three survey cycles: 2007–2008, 2009–2010, and 2011–2012.

**Figure 2:**
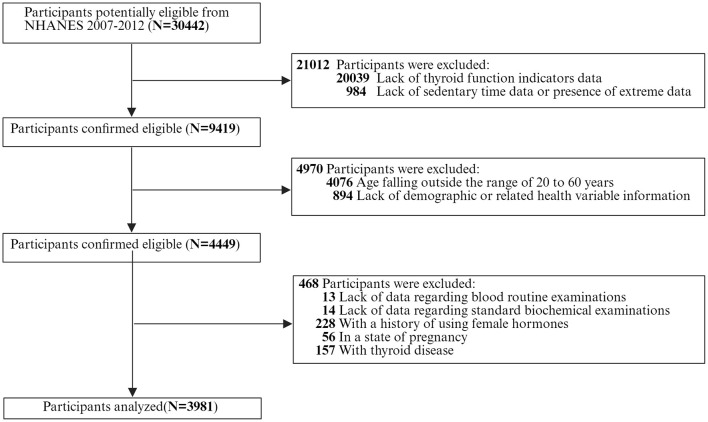
Participant flow diagram.

Initially, a total of 30,442 participants were enrolled in this study. We excluded the following individuals: (1) participants with missing thyroid function indices (*n* = 20,039); (2) participants with missing data on sedentary time or those with extreme values of sedentary time data (*n* = 984); (3) participants aged over 60 years old (*n* = 4,076), as the characteristics of thyroid function change significantly in the elderly population (34, 35); (4) participants with missing demographic or relevant health variables (*n* = 894); and (5) participants with missing results of routine blood tests (*n* = 13), standard biochemical tests (*n* = 14), those who were taking estrogen (*n* = 228), pregnant women (*n* = 56), or individuals with thyroid diseases (*n* = 157). Ultimately, 3,981 participants were included (Figure 2).

### Sedentary time

In the NHANES, the physical activity questionnaire is derived from the Global Physical Activity Questionnaire and encompasses a range of queries pertaining to daily activities, leisure pursuits, and sedentary behaviors. The sedentary time considered in this study excludes sleep duration and specifically pertains to activities such as sitting at a desk for occupational purposes, socializing with friends, commuting by car, bus, or train, reading, playing cards, watching television, and using a computer.

### Thyroid hormone sensitivity indicator

Thyroid hormone sensitivity indices were calculated by PTFQI, TSHI, and TT4RI. During the physical examination, serum FT4 levels were quantified using a two-step enzyme immunoassay, while serum TSH levels were measured using a third-generation two-site immunoenzymatic assay. In order to provide an index that can be calculated for any new value or adapted to other populations, an approximation with the same range and interpretation, the PTFQI can be obtained from FT4 in pmol/L and TSH in mIU/L using the standard normal cumulative distribution function as follows: Φ ((FT4 – μ FT4)/σ FT4) – (1 – Φ ((ln TSH – μln TSH)/σln TSH)), where μ FT4 = 10.075, σ FT4 = 2.155, μln TSH = 0.4654, and σln TSH = 0.7744 for the U.S. population (28). The PTFQI score exhibits a negative correlation with thyroid hormone sensitivity, indicating that an increase in the PTFQI score corresponds to a decrease in thyroid hormone sensitivity. TSHI was calculated as ln TSH (mIU/L) + 0.1345 × FT4 (pmol/L) (5). TT4RI was calculated as FT4 (pmol/L) × TSH (mIU/L) (36). The indices PTFQI, TSHI, and TT4RI are all constructed based on mathematical models of TSH and FT4 levels. Notably, PTFQI demonstrates more outstanding performance in terms of sensitivity and stability (9, 10).

### Covariates

In this study, covariates encompass demographic information, pertinent health variables, and laboratory test data. The demographic information includes gender, age, race, educational attainment (categorized as ≤ high school or >high school), marital status, and the poverty income ratio (PIR). Pertinent health variables comprise body mass index (BMI), smoking status, drinking status, and the presence of hypertension and diabetes. Smoking status is classified into smokers and non-smokers based on whether an individual has smoked more than 100 cigarettes in their lifetime. Drinking status is categorized into drinkers and non-drinkers according to whether a person has consumed more than 12 alcoholic beverages within 1 year. In this study, a diagnosis of diabetes is established if any of the following criteria are satisfied: (1) glycated hemoglobin A1c level ≥6.5% (47.5 mmol/mol); (2) fasting plasma glucose level ≥126 mg/dl (7.0 mmol/L); (3) random plasma glucose ≥200 mg/dl (11.1 mmol/L); (4) blood glucose level in a 2-h oral glucose tolerance test ≥200 mg/dl (11.1 mmol/L); (5) use of hypoglycemic drugs; and (6) participants with a self-reported diabetes diagnosis (37). Hypertension is diagnosed if any of the following criteria are met: (1) a medical professional has informed the individual of a hypertension diagnosis; (2) the use of antihypertensive medications; or (3) a systolic blood pressure of ≥140 mmHg or a diastolic blood pressure of ≥90 mmHg. Moderate-to-vigorous physical activity (MVPA) is composed of four indicators, namely vigorous recreational activities, moderate recreational activities, high-intensity work, and moderate-intensity work. Laboratory examination data included complete blood count and standard biochemistry tests.

### Statistical analysis

In accordance with the analytical guidelines set forth by the National Center for Health Statistics (NCHS), the complex survey design elements of the National Health and Nutrition Examination Survey (NHANES), such as weighting, clustering, and stratification, were meticulously considered. Data conforming to either a normal or skewed distribution were reported as “mean ± standard deviation”, while categorical variables were represented by the number of subjects and their respective percentages. The data were stratified into four quartile intervals based on sedentary time: Q1 (≤ 2 h), Q2 (2 < Q2 ≤ 4 h), Q3 (4 < Q3 ≤ 6 h), and Q4 (>6 h). The LASSO regression and the Boruta algorithm were used to screen covariates, aiming to identify the confounding factors that were closely associated with sedentary time and PTFQI. Multiple linear regression analysis and RCS analysis were employed to explore the association between sedentary time and PTFQI. In addition, subgroup analyses were conducted for categories such as age, sex, and body mass index (BMI), with all covariates except the variable of interest itself being adjusted. An interaction term was incorporated to test the heterogeneity of these associations. A two-sided *P* value threshold of < 0.05 was considered statistically significant. All analyses were performed using R Studio (version 4.4.2, United States).

## Results

### Baseline characteristics of participants

This study analyzed data from 3,981 participants drawn from the NHANES conducted in the United States between 2007 and 2012. Participants' baseline characteristics were categorized into four quartiles based on sedentary time (Table 1). Among the different quartile groups of sedentary time, significant differences were observed in sex, educational level, the ratio of family poverty to income (PIR), the prevalence of hypertension, BMI, TT4RI, TSHI, and PTFQI (all *P* values < 0.05). As sedentary time increased, TT4RI, TSHI, and PTFQI showed an upward trend, suggesting that sedentary behavior may have an adverse impact on thyroid hormone sensitivity.

**Table 1:** Baseline characteristics of the participants.

**Characteristic**	**Overall (*n* = 3,981)**	**Q1 (*n* = 1,016)**	**Q2 (*n* = 1,152)**	**Q3 (*n* = 819)**	**Q4 (*n* = 994)**	***P* value**
**Gender**, ***n*** **(%)**						
Male	2,189 (56%)	546 (55%)	693 (61%)	455 (54%)	495 (52%)	**0.001**
Female	1,792 (44%)	470 (45%)	459 (39%)	364 (46%)	499 (48%)	
**Age, Mean (95% CI)**	39 (29, 49)	39 (30, 48)	38 (29, 49)	40 (29, 50)	40 (30, 50)	0.541
**Race**, ***n*** **(%)**						
Non-Hispanic Whites	1,695 (66%)	316 (54%)	506 (67%)	396 (71%)	477 (71%)	**< 0.001**
Non-Hispanic Black	792 (11%)	187 (13%)	214 (10%)	171 (11%)	220 (11%)	
Mexican American	752 (9.9%)	301 (19%)	240 (11%)	102 (6.1%)	109 (4.9%)	
Others	742 (13%)	212 (14%)	192 (12%)	150 (11%)	188 (13%)	
**Education**, ***n*** **(%)**						
≤ High school	1,997 (42%)	681 (60%)	625 (47%)	363 (38%)	328 (27%)	**< 0.001**
>High school	1,984 (58%)	335 (40%)	527 (53%)	456 (62%)	666 (73%)	
**Marital status**, ***n*** **(%)**						
Married or cohabiting	2,413 (63%)	651 (66%)	730 (64%)	458 (61%)	574 (62%)	0.111
Living alone	604 (13%)	166 (15%)	165 (13%)	128 (13%)	145 (13%)	
Single	964 (24%)	199 (19%)	257 (22%)	233 (26%)	275 (25%)	
**PIR**, ***n*** **(%)**						
≤ 1.0	973 (17%)	306 (25%)	285 (17%)	203 (18%)	179 (12%)	**< 0.001**
1.0–3.0	1,607 (34%)	483 (43%)	477 (36%)	309 (34%)	338 (27%)	
≥3.0	1,401 (49%)	227 (33%)	390 (47%)	307 (49%)	477 (61%)	
**Smoking**, ***n*** **(%)**						
Yes	1,783 (45%)	435 (47%)	526 (47%)	385 (45%)	437 (43%)	0.686
No	2,198 (55%)	581 (53%)	626 (53%)	434 (55%)	557 (57%)	
**Drinking**, ***n*** **(%)**						
Yes	3,057 (81%)	728 (76%)	897 (81%)	636 (81%)	796 (84%)	**0.015**
No	924 (19%)	288 (24%)	255 (19%)	183 (19%)	198 (16%)	
**Hypertension**, ***n*** **(%)**						
Yes	1,039 (24%)	252 (22%)	269 (21%)	227 (26%)	291 (29%)	**< 0.001**
No	2,942 (76%)	764 (78%)	883 (79%)	592 (74%)	703 (71%)	
**Diabetes**, ***n*** **(%)**						
Yes	455 (8.6%)	120 (8.3%)	109 (7.3%)	104 (9.1%)	122 (9.7%)	0.418
No	3,526 (91%)	896 (92%)	1,043 (93%)	715 (91%)	872 (90%)	
BMI, Mean (95% CI)	27 (24, 32)	27 (24, 31)	27 (24, 31)	28 (24, 32)	28 (24, 32)	**0.025**
TT4RI, Mean (95% CI)	15 (10, 22)	14 (9, 22)	15 (10, 22)	15 (11, 22)	16 (11, 23)	**0.020**
TSHI, Mean (95% CI)	1.77 (1.38, 2.17)	1.68 (1.29, 2.15)	1.77 (1.38, 2.19)	1.80 (1.41, 2.15)	1.81 (1.41, 2.19)	**0.015**
PTFQI, Mean (95% CI)	−0.03 (−0.24, 0.19)	−0.06 (−0.28, 0.14)	−0.05 (−0.25, 0.18)	0.00 (−0.24, 0.19)	0.00 (−0.21, 0.21)	**0.001**

For categorical variables, the P value was determined using the weighted chi-square test. For continuous variables, data are presented as mean ± standard error (SE), and the P value was calculated using the weighted Kruskal–Wallis test. Bolded values indicate statistically significant differences. Sedentary time was divided into four quartile intervals: Q1 ( ≤ 2 h), Q2 (2 < Q2 ≤ 4 h), Q3 (4 < Q3 ≤ 6 h), and Q4 (>6 h).

PIR, Poverty Income Ratio; BMI, body mass index; PTFQI: parametric thyroid feedback quantile-based index; TSHI, thyroid-stimulating hormone index; TT4RI, thyrotrophin thyroxine resistance index.

### Screening of confounding variables

**Figure 3:**
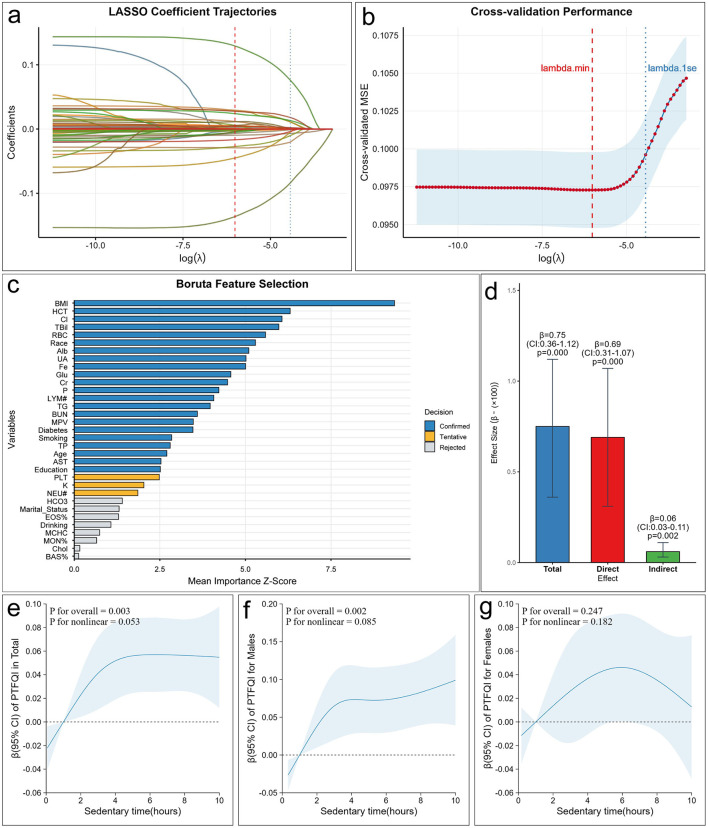
Confounder selection and the association between sedentary time and thyroid hormone sensitivity. **(a)** LASSO Coefficient distribution map for all confounding variables; **(b)** variables determined by LASSO analysis; **(c)** feature importance of confounding variables selected by LASSO regression assessed using the Boruta algorithm; **(d)** mediating effect of BMI on the association between sedentary time and PTFQI; **(e)** RCS curve of sedentary time and PTFQI for all participants; **(f)** RCS curve of sedentary time and PTFQI for male participants; **(g)** RCS curve of sedentary time and PTFQI for female participants. BMI, body mass index; Hb, hemoglobin; Cl, chloride; TBil, total bilirubin; RBC, red blood cell count; Alb, Albumin; UA, uric acid; Fe, Iron; Glu, serum Glucose; Cr, creatinine; P, phosphorus; LYM#, lymphocyte number; TG, triglyceride; BUN, blood urea nitrogen; MPV, mean platelet volume; TP, total protein; AST, aspartate aminotransferase; PLT, platelet count; K, potassium; NEU#, segmented neutrophils number; HCO3, bicarbonate; EOS%, eosinophils percent; MCHC, mean corpuscular hemoglobin concentration; MON%, monocyte percent; Chol, cholesterol; BAS%, basophils percent; PTFQI, parametric thyroid feedback quantile-based index.

In this study, the LASSO regression and the Boruta machine learning algorithm were utilized to systematically identify the potential confounding variables between sedentary time and thyroid hormone sensitivity. In the LASSO regression analysis, the minimum lambda value was determined through 10-fold cross-validation. This lambda value achieved a balance between model complexity and prediction error, effectively preventing the model from being overfitted or oversimplified (Figures 3a, b).

Subsequently, the covariates screened out by the LASSO regression were further incorporated into the unweighted Boruta algorithm analysis. The Boruta algorithm evaluates the importance of features by introducing shadow variables, thus precisely identifying the covariates that have a significant impact on the dependent variable. After 200 iterations, the Boruta algorithm screened out 22 confounding factors of significant importance, which were included in the subsequent study (Figure 3c). It is worth noting that BMI ranked first among the importance scores of these features. Further mediation analysis revealed that BMI played a mediating role in the association between sedentary time and PTFQI (β = 0.0006, 95% CI: 0.0003–0.0011), and the proportion of the mediating effect was 7.99% (Figure 3d).

### Association between thyroid hormone sensitivity and sedentary time

The scatter plots and linear fitting curves demonstrated direct links between sedentary time and thyroid hormone-related indicators such as PTFQI, TSHI, TT4RI, and TSH (Supplementary Figure 1). By using three times the standard deviation as a threshold, it was found that TSHI, TT4RI, and TSH had more outliers compared to PTFQI. These outliers suggested that the relationships between sedentary time and TSHI, TT4RI, and TSH might contain abnormal data that could influence the results, whereas PTFQI was less affected by outliers.

In Table 2, it is demonstrated that, after adjusting for all confounding variables in Model 3, the PTFQI level in the highest quartile among men was significantly elevated compared to that in the lowest quartile (β = 0.09, 95% CI: 0.04–0.13, *P* < 0.001, *P* for trend < 0.001). Supplementary Table 1 illustrates that this association persisted even when MVPA was included as a covariate in Model 3 (β = 0.07, 95% CI: 0.00–0.14, *P* = 0.043, *P* for trend = 0.034). Conversely, in women, the association between sedentary time and PTFQI was not statistically significant (all *P* values > 0.05). Following adjustment for all covariates, restricted cubic spline (RCS) analysis was conducted to investigate the potential non-linear relationship between sedentary time and thyroid hormone sensitivity. In the total population, the curve depicting the association between sedentary time and PTFQI initially rose and then leveled off (Figure 3e, *P* for overall = 0.003, *P* for non-linear = 0.053). The findings indicate a significant linear association between sedentary time and PTFQI among men (Figure 3f, *P* for overall = 0.002, *P* for non-linear = 0.085). In contrast, an “inverted U-shaped” relationship was observed among women, although this relationship did not reach statistical significance (Figure 3g, *P* for overall = 0.247). Supplementary Figure 2 illustrates the non-linear relationship between sedentary time and TSHI in male participants (*P* for overall = 0.012, *P* for non-linear = 0.038), whereas no statistically significant association was observed in female participants.

**Table 2:** Correlation between sedentary time and PTFQI.

**Characteristic**	**Model 1**		**Model 2**		**Model 3**	
	β **(95% CI)**	***P*** **-value**	β **(95% CI)**	***P*** **-value**	β **(95% CI)**	***P*** **-value**
**Overall**						
Q1	Ref	Ref	Ref	Ref	Ref	Ref
Q2	0.03 (0.00, 0.06)	**0.049**	0.03 (−0.00, 0.06)	0.091	0.03 (−0.00, 0.06)	0.072
Q3	0.05 (0.01, 0.08)	**0.014**	0.04 (0.01, 0.08)	**0.025**	0.04 (0.01, 0.08)	**0.021**
Q4	0.07 (0.04, 0.11)	**< 0.001**	0.06 (0.03, 0.10)	**< 0.001**	0.06 (0.02, 0.09)	**0.003**
*P* trend		**< 0.001**		**< 0.001**		**< 0.001**
**Male**						
Q1	Ref	Ref	Ref	Ref	Ref	Ref
Q2	0.03 (0.00, 0.07)	0.053	0.03 (−0.00, 0.07)	0.078	0.04 (0.00, 0.07)	**0.037**
Q3	0.04 (−0.00, 008)	0.057	0.03 (0.01, 0.07)	0.088	0.04 (0.00, 0.08)	0.051
Q4	0.10 (0.05, 0.15)	**< 0.001**	0.09 (0.05, 0.14)	**< 0.001**	0.09 (0.04, 0.13)	**< 0.001**
*P* trend		**< 0.001**		**< 0.001**		**< 0.001**
**Female**						
Q1	Ref	Ref	Ref	Ref	Ref	Ref
Q2	0.02 (−0.02, 007)	0.293	0.02 (−0.03, 0.06)	0.415	0.02 (−0.02, 0.06)	0.352
Q3	0.05 (−0.01, 0.10)	0.096	0.04 (−0.01, 0.10)	0.128	0.04 (−0.02, 0.09)	0.165
Q4	0.05 (−0.02, 0.11)	0.160	0.03 (−0.03, 0.10)	0.301	0.03 (−0.03, 0.09)	0.265
*P* trend		0.143		0.252		0.263

Model 1 did not adjust for confounding factors. Model 2 was adjusted according to demographic and relevant health variables, including age, race, education, smoking, diabetes and BMI. Model 3 was adjusted according to demographic information, relevant health variables, and laboratory test variables, including age, race, education, smoking, diabetes, BMI, hematocrit, serum chloride, total bilirubin, red blood cell count, albumin, uric acid, serum iron, serum glucose, serum creatinine, phosphorus, lymphocyte count, triglycerides, blood urea nitrogen, mean platelet volume, total protein and aspartate aminotransferase. Bolded values indicate statistically significant differences. The P trend was obtained by converting sedentary time from a continuous variable to an ordinal categorical variable (Q1, Q2, Q3, and Q4) and performing regression analysis using the median of each category.

PTFQI, parametric thyroid feedback quantile-based index.

### Analysis of subgroups and interaction tests

**Figure 4:**
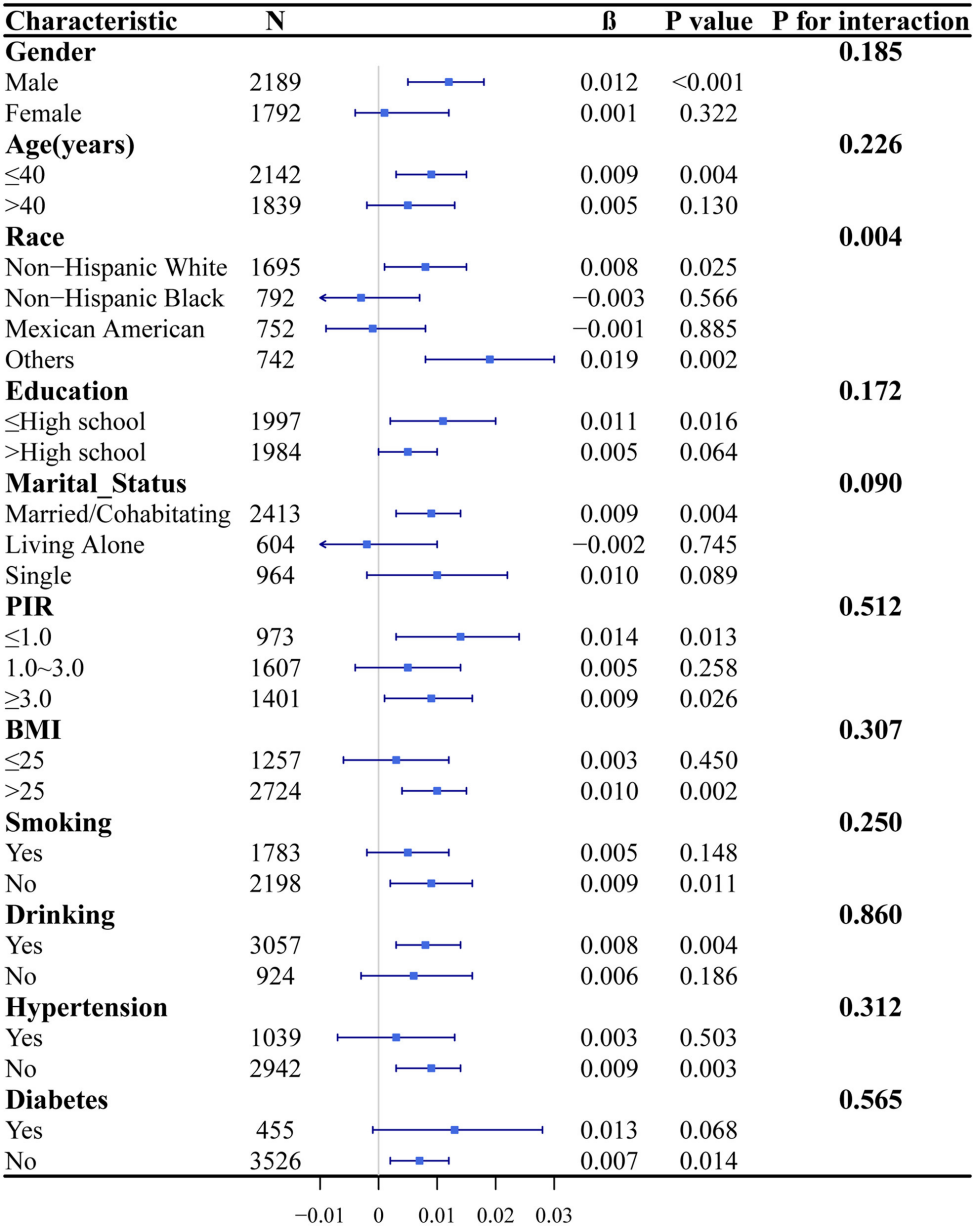
Subgroup analyses and interaction tests between sedentary time and PTFQI. In subgroup analyses, all covariates were adjusted, with the exception of the variable of interest.

In Figure 4, subgroup analyses were performed to explore the associations between sedentary time and PTFQI levels across diverse populations stratified by gender, age, race, educational attainment, marital status, PIR, BMI, smoking status, alcohol consumption, hypertension, and diabetes. The analysis revealed a significant positive correlation between sedentary time and PTFQI within subgroups such as males and individuals with obesity. Furthermore, interaction analysis demonstrated that race significantly moderated the association between sedentary time and PTFQI (*P* for interaction = 0.004). Specifically, a positive correlation was identified among non-Hispanic whites and other ethnic groups, whereas this association was not significant among non-Hispanic blacks and Mexican Americans.

## Discussion

The association between thyroid health and sedentary behavior represents a relatively under-explored area in current research, warranting further investigation. Thyroid hormones, as crucial endocrine hormones, can regulate the body's energy metabolism, influence carbohydrate, protein, and fat metabolism, and participate in cardiovascular regulation (38, 39). The reduced energy expenditure and metabolic deceleration associated with sedentary behavior may disrupt the normal functioning of thyroid hormones, thereby impacting the body's metabolic state and energy equilibrium (40, 41). Furthermore, there is a significant positive correlation between sedentary behavior and elevated levels of C-reactive protein, abnormal lipid metabolism, insulin resistance index, and insulin concentration (42). These metabolic and inflammatory factors are likely linked to thyroid dysfunction (43). A Mendelian randomization study by Zhang et al. (44) found that higher genetically-influenced TSH levels are linked to increased leisure screen time. Additionally, an epidemiological study showed that sitting for over 6 h daily raises the risk of 12 chronic diseases, including thyroid issues, by 26%. Reducing sedentary time to under 6 h could prevent 3.7%−22.1% of these diseases (45). Common diseases linked to both sedentary behavior and impaired thyroid sensitivity include cardiovascular diseases, cancer, and metabolic syndrome, among others (Figure 1). However, there is currently a lack of direct evidence to definitively establish the correlation between these factors.

In this study, we integrated the LASSO regression and the Boruta algorithm to establish a dual feature screening framework. The LASSO regression, through L1 regularization, achieved efficient dimensionality reduction, meticulously selecting core covariates to form a parsimonious variable set. The Boruta algorithm, relying on the random forest mechanism, generated shadow variables to thoroughly uncover non-linear relationships and interaction effects among variables. This approach effectively circumvented the inherent flaws of path dependence and multicollinearity in stepwise regression, while also overcoming the limitations of the linear assumptions in LASSO regression. The synergistic application of these two methods established a robust system for controlling confounding factors in the exploration of the association between sedentary time and thyroid hormone sensitivity.

Among the numerous confounding factors involved in this study, BMI took the primary position, and BMI partially mediated the association between sedentary time and the PTFQI. This phenomenon may be attributed to several factors. Firstly, previous research has demonstrated an intrinsic link between thyroid hormone resistance and obesity, with this resistance being particularly pronounced in obese individuals (46). Secondly, BMI is closely associated with various metabolic parameters, including insulin resistance and dyslipidemia. Alterations in these metabolic parameters may potentially influence the mechanism of action of thyroid hormones (47). Thirdly, BMI may also indirectly affect thyroid hormone sensitivity by influencing the body's inflammatory state. Generally, obesity is often accompanied by chronic low-grade inflammation, and this inflammatory state is highly likely to alter the metabolic process and action effect of thyroid hormones (48).

The integrated examination of sedentary behavior and physical activity has been extensively utilized in the investigation of various chronic diseases (49, 50). The influence of physical activity on thyroid function is complex, with distinct effects observed between occupational physical activity and leisure-time exercise. Research indicates that occupational physical activity is correlated with a reduction in thyroid function and an increase in thyroid autoimmunity, whereas leisure-time exercise is associated with decreased TSH levels (51). Tian et al. (52) have demonstrated that the daily physical activity levels of American adults are significantly linked to modifications in thyroid function, including variations in thyroid hormone levels and the prevalence of thyroid disorders. Furthermore, the impact of exercise on thyroid function may also be mediated by myokines secreted by muscles (53).

In the investigation of the relationship between sedentary behavior and thyroid hormone sensitivity, a significant positive correlation was identified in males, whereas no significant association was detected in females. Despite both genders possessing the hypothalamic-pituitary-thyroid (HPT) axis to regulate thyroid hormone secretion, there are notable gender-specific differences in the regulatory mechanisms. For instance, during distinct physiological phases such as the menstrual cycle and pregnancy, women experience adaptive changes in the HPT axis (54). Studies indicate that thyroid hormone levels are associated with variations in the concentrations of sex hormone-binding globulin and albumin, which may manifest differently in males and females (55). Furthermore, disparities in lifestyle and environmental factors may also influence thyroid hormone sensitivity across genders (56, 57). The interaction effect of race on the relationship between sedentary time and PTFQI may be attributed to genetic variations within the thyroid hormone metabolic pathway, suggesting that genetic factors contribute to differences in thyroid hormone sensitivity among various racial groups (58).

In this study, we identified several key limitations that may impact the interpretation and generalizability of our findings. Firstly, the cross-sectional design of our study constrains our ability to establish a causal relationship between sedentary behavior and thyroid hormone sensitivity. For instance, individuals with hypothyroidism may exhibit a reduction in metabolic rate and sympathetic excitability, potentially influencing their sedentary behavior (59). Secondly, a significant and challenging source of bias arises from the possibility that participants reporting a sedentary lifestyle may decrease their physical activity due to undiagnosed or subclinical conditions (44). Lastly, the data on sedentary time were obtained through self-reported questionnaires, introducing a degree of subjectivity. Future research should consider employing more objective methods to measure sedentary time, thereby minimizing measurement errors and other biases associated with self-reported recall.

## Conclusion

In this study, we found a positive association between impaired thyroid hormone sensitivity and sedentary time in men after adjusting for confounders, and BMI partially mediated this positive association. Additionally, the factor of race exhibited a significant interaction effect on the association between sedentary time and the PTFQI.

## Data Availability

Publicly available datasets were analyzed in this study. This data can be foundaspx at: https://wwwn.cdc.gov/nchs/nhanes/Default.
