# Supplementary material for: Association between impaired sensitivity to thyroid hormones and sedentary behavior: a cross-sectional study
Source: Front Med (Lausanne). 2025 Jun 6;12:1596669. doi: 10.3389/fmed.2025.1596669 (PMC12179160; doi:10.3389/fmed.2025.1596669)
Supplement: Supplementary file 1 [file Data_Sheet_1.docx]

**Supplementary materials**

**Supplementary Figure 1.** Scatter plots of sedentary time and indicators of thyroid hormone sensitivity.

**Supplementary Figure 2.** RCS curves of sedentary time and TSH, TSHI, TT4RI.

**Supplementary Table 1.** The association between sedentary time and PTFQI after adjusting for MVPA.


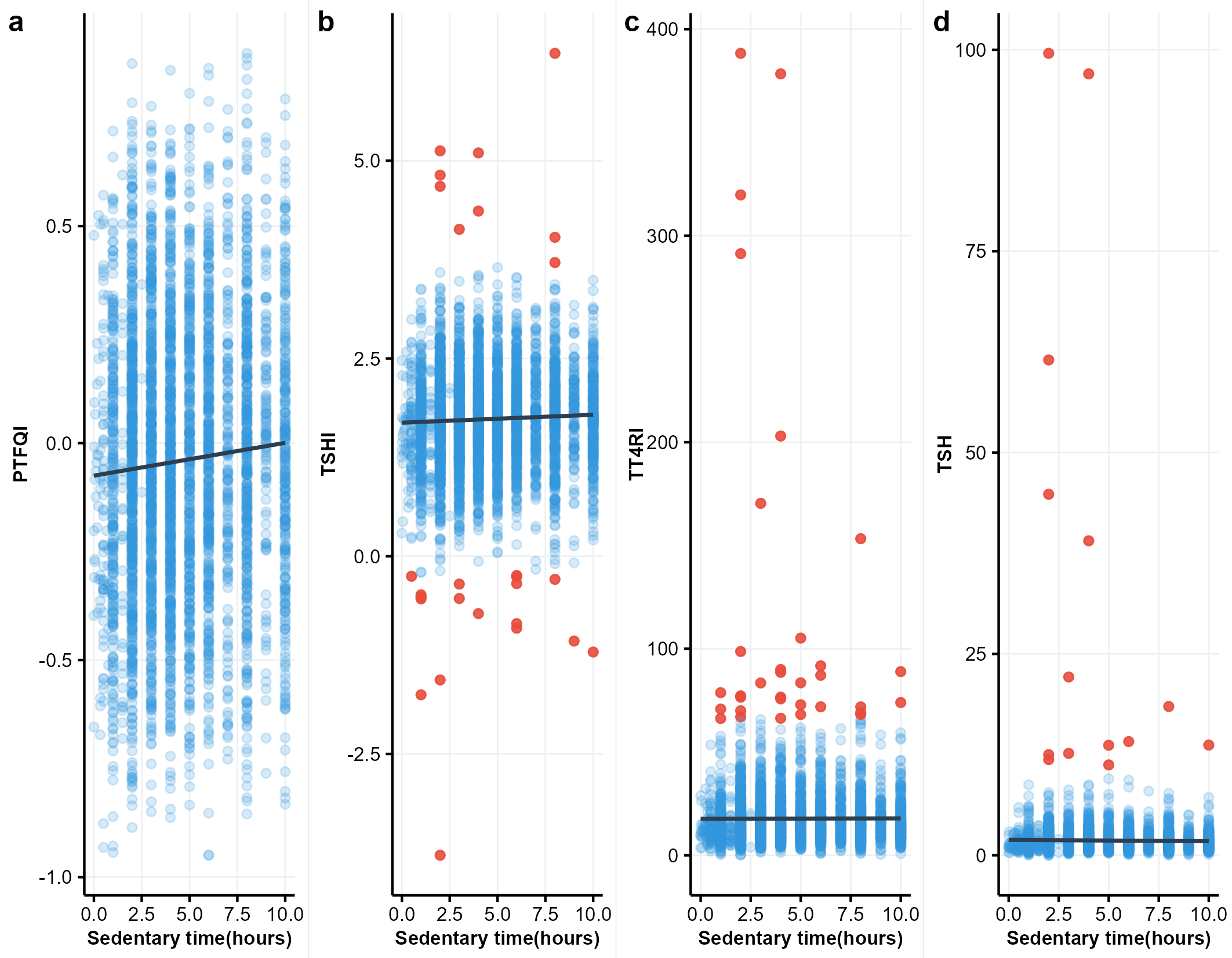
**Supplementary Figure 1.** Scatter plots of sedentary time and indicators of thyroid hormone sensitivity.

(a) Scatter plot of sedentary time and PTFQI; (b) scatter plot of sedentary time and TSHI; (c) scatter plot of sedentary time and TT4RI; (d) scatter plot of sedentary time and TSH. The black lines represent the linear fit curves, and the red dots indicate outliers beyond three times the standard deviation. Abbreviations: PTFQI, parametric thyroid feedback quantile-based index; TSHI, thyroid-stimulating hormone index; TT4RI, thyrotrophin thyroxine resistance index; TSH, **Thyroid-stimulating hormone.**


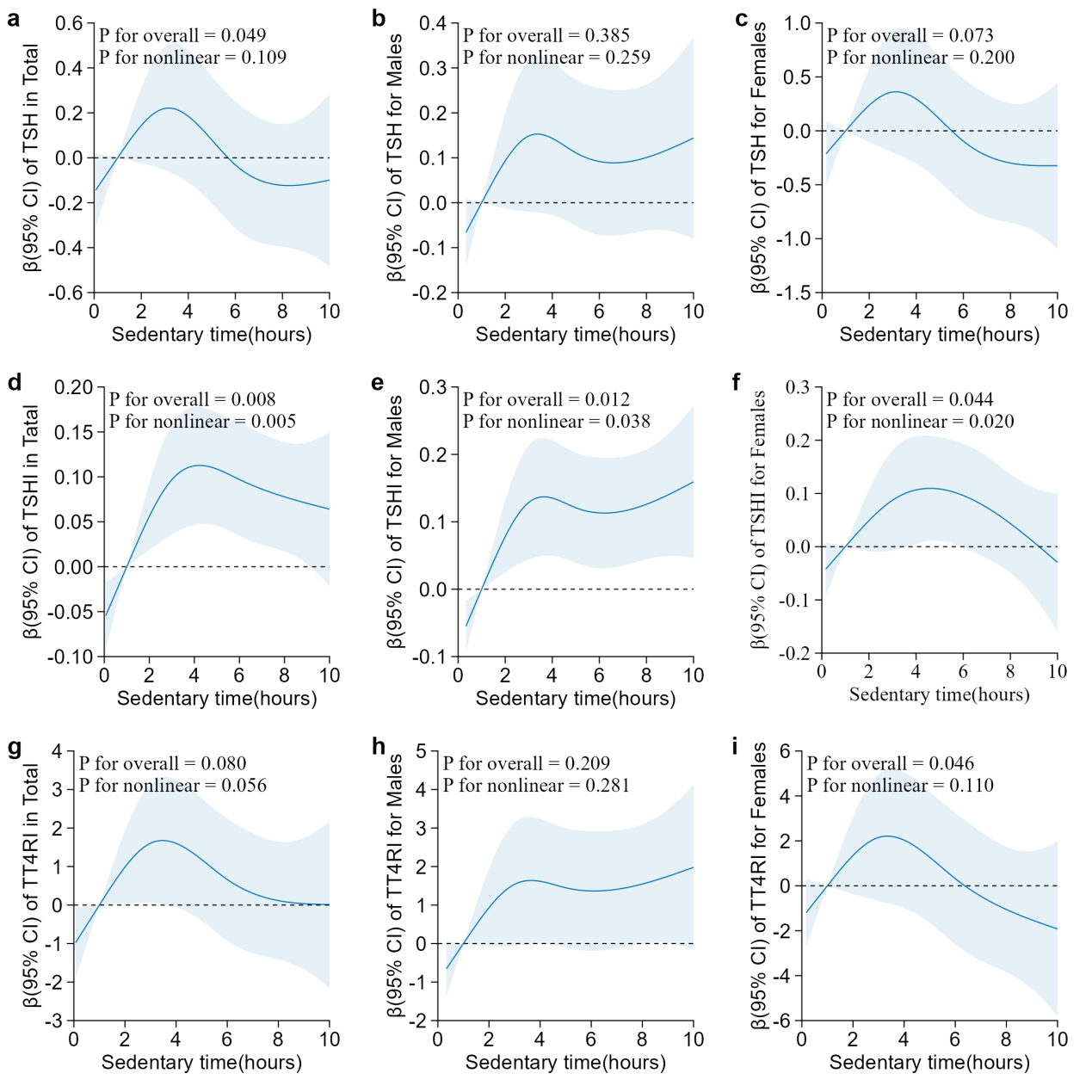


**Supplementary Figure 2.** RCS curves of sedentary time and TSH, TSHI, TT4RI.

Figures a to i respectively show the gender differences in the non-linear associations between sedentary time and TSH, TSHI, TT4RI. Abbreviations: PTFQI, parametric thyroid feedback quantile-based index; TSHI, thyroid-stimulating hormone index; TT4RI, thyrotrophin thyroxine resistance index; TSH, Thyroid-stimulating hormone; TSH, Thyroid-stimulating hormone.

**Supplementary Table 1.** The association between sedentary time and PTFQI after adjusting for MVPA.

| Characteristic | Model1 | | Model2 | | Model3 | |
| --- | --- | --- | --- | --- | --- | --- |
|  | β(95%CI) | p | β(95%CI) | p | β(95%CI) | p |
| Overall |  |  |  |  |  |  |
| Q1 | Ref | Ref | Ref | Ref | Ref | Ref |
| Q2 | -0.01(-0.06,0.04) | 0.622 | -0.01(-0.06,0.04) | 0.645 | -0.01(-0.06,0.05) | 0.745 |
| Q3 | 0.01(-0.03,0.06) | 0.515 | 0.01(-0.03,0.05) | 0.720 | 0.00(-0.04,0.05) | 0.932 |
| Q4 | 0.05(0.00,0.10) | **0.040** | 0.04(-0.01,0.09) | 0.128 | 0.03(-0.02,0.08) | 0.178 |
| P trend |  | **0.033** |  | 0.115 |  | 0.186 |
| Male |  |  |  |  |  |  |
| Q1 | Ref | Ref | Ref | Ref | Ref | Ref |
| Q2 | -0.00(-0.06,0.05) | 0.933 | 0.00(-0.05,0.06) | 0.982 | 0.00(-0.05,0.06) | 0.878 |
| Q3 | 0.03(-0.02,0.09) | 0.195 | 0.03(-0.03,0.08) | 0.338 | 0.02(-0.04,0.08) | 0.492 |
| Q4 | 0.09(0.02,0.16) | **0.010** | 0.08(0.01,0.15) | **0.035** | 0.07(0.00,0.14) | **0.043** |
| P trend |  | **0.005** |  | **0.024** |  | **0.034** |
| Female |  |  |  |  |  |  |
| Q1 | Ref | Ref | Ref | Ref | Ref | Ref |
| Q2 | -0.02(-0.09,0.04) | 0.464 | -0.02(-0.08,0.04) | 0.528 | -0.03(-0.09,0.03) | 0.266 |
| Q3 | -0.01(-0.07,0.06) | 0.864 | 0.04(-0.06,0.07) | 0.904 | -0.01(-0.07,0.06) | 0.864 |
| Q4 | 0.01(-0.07,0.08) | 0.984 | -0.01(-0.09,0.07) | 0.810 | -0.01(-0.08,0.07) | 0.869 |
| P trend |  | 0.810 |  | 0.996 |  | 0.842 |

On the basis of the original 3,981 participants, those with missing exercise data were excluded. Finally, 2,045 participants were included in this linear regression model. Model 1 did not adjust for confounding factors. Model 2 was adjusted according to demographic and relevant health variables, including age, race, education, smoking, diabetes and BMI. Model 3 was adjusted according to demographic information, relevant health variables, and laboratory test variables, including age, race, education, smoking, diabetes, BMI, hematocrit, serum chloride, total bilirubin, red blood cell count, albumin, uric acid, serum iron, serum glucose, serum creatinine, phosphorus, lymphocyte count, triglycerides, blood urea nitrogen, mean platelet volume, total protein, aspartate aminotransferase and MVPA. Bolded values indicate statistically significant differences. The P trend was obtained by converting sedentary time from a continuous variable to an ordinal categorical variable (Q1, Q2, Q3, Q4) and performing regression analysis using the median of each category. Abbreviations: PTFQI, parametric thyroid feedback quantile-based index; MVPA, Moderate-to-vigorous physical activity.
